# Comparison of clinical examinations of back disorders and humans’ evaluation of back pain in riding school horses

**DOI:** 10.1186/1746-6148-9-209

**Published:** 2013-10-15

**Authors:** Clémence Lesimple, Carole Fureix, Véronique Biquand, Martine Hausberger

**Affiliations:** 1 Université de Rennes1, UMR CNRS 6552 Ethologie Animale et Humaine 263 avenue du général Leclerc, Rennes cedex 35042, France

**Keywords:** Horses, Welfare, Back disorders, Practitioner evaluation, Static electromyographic measures, Questionnaire

## Abstract

**Background:**

Questionnaires are a common tool to assess people’s opinion on a large scale or to sound them out about their subjective views. The caretakers’ opinion about animals’ “personality” has been used in many studies. The aim of the present study was to assess whether the owners’ subjective evaluation was effective to detect back disorders. Back disorders have been shown to have a high prevalence in working horses. Caretakers from 17 riding schools (1 caretaker/school, 161 horses) were given a questionnaire about their horses’ health status, including back disorders. Out of these 161 horses, 59 were subjected to manual palpation of the spine and 102 were subjected to sEMG examination all along the spine.

**Results:**

The results showed that subjective caretaker-reported evaluation via questionnaire survey was not efficient to detect back disorders: only 19 horses (11.8%) were reported as suffering from back pain, whereas the experimenters’ evaluation detected 80 of them (49.7%) as suffering from back disorders. While most caretakers under-evaluated back disorders, a few “over-evaluated” it (more horses reported as affected than found via clinical evaluations). Horses were less prone to present back disorders when under the care of these “over-attentive” caretakers.

**Conclusions:**

This study showed that back pain is difficult to evaluate, even for professionals, and that subjective evaluations using a questionnaire is not valid in this case. The results also highlighted the real need for observational training (behaviours, postures) outside and during riding.

## Background

Back disorders are recognized as a common problem in working horses [1-5]. The estimated prevalence varies from 27% [4] to 100% [1] of the ridden horse population. Back disorders are difficult to detect on the basis of the behaviour [6] and to evaluate objectively in the field on large samples of horses whether by radiographic, ultrasonic or scintigraphic imaging [7,8]. As a consequence, horses often continue to be used in athletic activities despite the discomfort/pain caused *e.g.*[9]. Evaluation of pain in domestic or laboratory animals by humans is difficult [10,11] especially in species such as horses *e.g.*[10]. Owners or caretakers may have personal interpretations of behaviours they assume to reflect discomfort or pain [6]. Apart from cases with overt associated lameness or gait alteration [4], horses mainly express back pain problems through progressive or sudden changes in temperament [7], such as an increased aggressiveness towards humans [4,12] signs of escape attempts *e.g.*[4] or particular postures at work, in which horses may try to escape back pain [13,14].

Although many authors have mentioned the underestimation of back disorders in working horses *e.g.*[1-4,7], no study has yet investigated the validity of subjective evaluation of back pain by people who work with them. If an owner’s or caretaker’s perception is unreliable, it is urgent that discrepancies between users’ evaluations and clinical evaluations of back disorders are highlighted and strategies to improve the detection proposed. The aim of the present study was to compare subjective evaluation by the daily caretakers with different methods of clinical evaluation of potential back pain problems. Since “classical” imaging techniques cannot be used in the field, we evaluated horses’ potential back problems with either a manual examination by a chiropractor, who as a licensed professional, has an expertise in the evaluation of spinal disorders [15,16] or with a technique increasingly used for detecting back problems in humans: static surface EMG (sEMG). Indeed sEMG measures have been shown to reflect various muscular dysfunctions and patients with lower back pain display higher static sEMG values [17-19]. In horses, sEMG values has only been used to explore muscular activity during movements [20,21] although we have been able to demonstrate that chiropractic and sEMG evaluations were correlated in a sample of horses at rest [22]. The caretakers’ opinion about the horses’ back status was measured using standardized questionnaires, an approach that has been used in earlier studies on animals’ personality [23,24] and behaviour [25]. More than 150 riding school horses were used for this study. Such horses are known to be prone to develop back disorders [12,14] while being maintained at work.

## Methods

### Animals

In each riding school, owners gave their oral consent to the experimenter for the horses’ back evaluations via manual palpation or sEMG measures. They worked in riding lessons involving children and teenagers for 4–20 hours per week, with at least 1 day resting. They were only used for teaching, with riders from beginner to intermediate levels. The horses’ age distribution did not differ between schools, neither in the first, nor in the second study (Kruskall-Wallis ANOVA (KW), respectively H(2, N = 59) = 1.5 and H(13, N = 101) = 14.2, p > 0.3 in each case).

#### Study 1: practitioner evaluation

The first study was performed in 2007 (see also [14,16]) on fifty-nine horses (44 geldings, 15 mares; 5–20 years old $\left[\bar{X}\pm \mathrm{se}=12.81\pm 0.46\right]$; mostly French saddlebreds: 68%, and smaller proportions of Connemara, French Trotters, Thoroughbreds and unregistered animals). The horses were evaluated both via manual palpation by a practitioner and with a questionnaire handed out to the daily caretakers. They were distributed across 3 riding schools (RS1, RS2 and RS3; 19.7 ± 4.9 horses per school) with similar activities and housing conditions. Thus, horses worked from Monday to Saturday (± 4 h/day) and were free on Sundays, with a maximal activity during school time (Monday to Friday). Horses were kept singly in straw-bedded individual boxes cleaned once a day, they were fed industrial pellets 3 times a day, hay once a day and had water *ad libitum*. All horses involved in the riding schools’ activities at the time of the study were included.

#### Study 2: static electromyographic evaluation

Since the first study gave interesting results, horses’ back problems were further investigated in a second larger study initiated in 2010 in riding schools. Given the constraints related to manual palpation (in particular the necessary presence of a practitioner all along the study) a new approach through static sEMG was chosen since it was easily transportable, adapted to the horse and it had proved efficient in the detection of back pain in humans [18,19]. A first study revealed that when applied to the same horse population, these two evaluation methods gave consistent results. The second study included measurements of other parameters concerning welfare issues (time spent in paddocks, working time and practices, feeding…) on a “traditional” type of management (Lesimple et al in prep.).

The sEMG evaluation was conducted on 102 horses (45 mares, 57 geldings), of varied ages (4-23 years $\left[\bar{X}\pm \mathrm{se}=13.3\pm 0.45\right]$), and breeds (N = 13, mostly unregistered horses: 44.11%, French ponies: 27.5%, French Saddlebreds: 16.5% and smaller proportions of Connemaras, Anglo-Arabians, Haflingers, Merens, French trotters, Throughbreds, Welsh ponies and Pottoks) coming from 14 riding schools all over France (7.3 ± 0.8 horses per school). All these horses were also evaluated using a questionnaire handed out to the daily caretakers. In each case, all horses involved in the riding schools’ activities at the time of the study were included. Horses had 2 full days of work on Wednesdays and Saturdays (± 4 h/day), and worked one to two hours per day during the rest of the week. All had at least one free day (usually Sunday). Horses were under the management of riding schools, mostly housed in straw bedded individual boxes/stalls (87.2%) or in individual (6.9%) or group (5.9%) pastures. Most of them were fed industrial pellets (87.3%), two (43.8%) or three times (48.3%) per day. Seven horses had pellets only once a day (7.9%) and 13 (12.7%) were not fed pellets. Most of them also had hay (only 6 had not, because of a lack of hay in the area) distributed in 1 to 5 meals. All horses had water ad libitum.

### Back health evaluation methods

#### Manual palpation

Evaluation of the horses’ spine was performed by a 20 years experienced licensed chiropractor (H. M), expert in the evaluation of joints and spinal related disorders [18,19], who was not familiar with any of the horses beforehand. Manual palpation was performed from head to tail and the mobility of each vertebral site was tested (N = 51 vertebral sites: 7 cervical, 18 thoracic, 6 lumbar, 5 sacral and 15 coccigeal). Examination was based on bony and soft tissue manual palpation to localise regions of vertebral stiffness based on spinal mobilisation and palpable areas of muscle hypertonicity [26,27]. Comparisons of data from different practitioners in earlier studies (including H.M involved here) have shown high agreement and therefore repeatability (*i.e.* 94% of the vertebrae [14,22]). Examinations were performed outside the horses’ working times, during the resting day, in each horse’s individual box. The examined horse was lightly restrained by one unknown (to the horses) experimenter (M.H). Horses were classified by the practitioner as totally unaffected, slightly affected (one affected vertebra) or severely affected (at least 2 affected vertebrae). Data included also the percentage of affected vertebrae per horse. As soon as a problem was detected at the level of a vertebra (stiffness or hypertonicity or both) the vertebral site was considered as affected.

#### Static surface electromyogram (sEMG)

**Figure 1:**
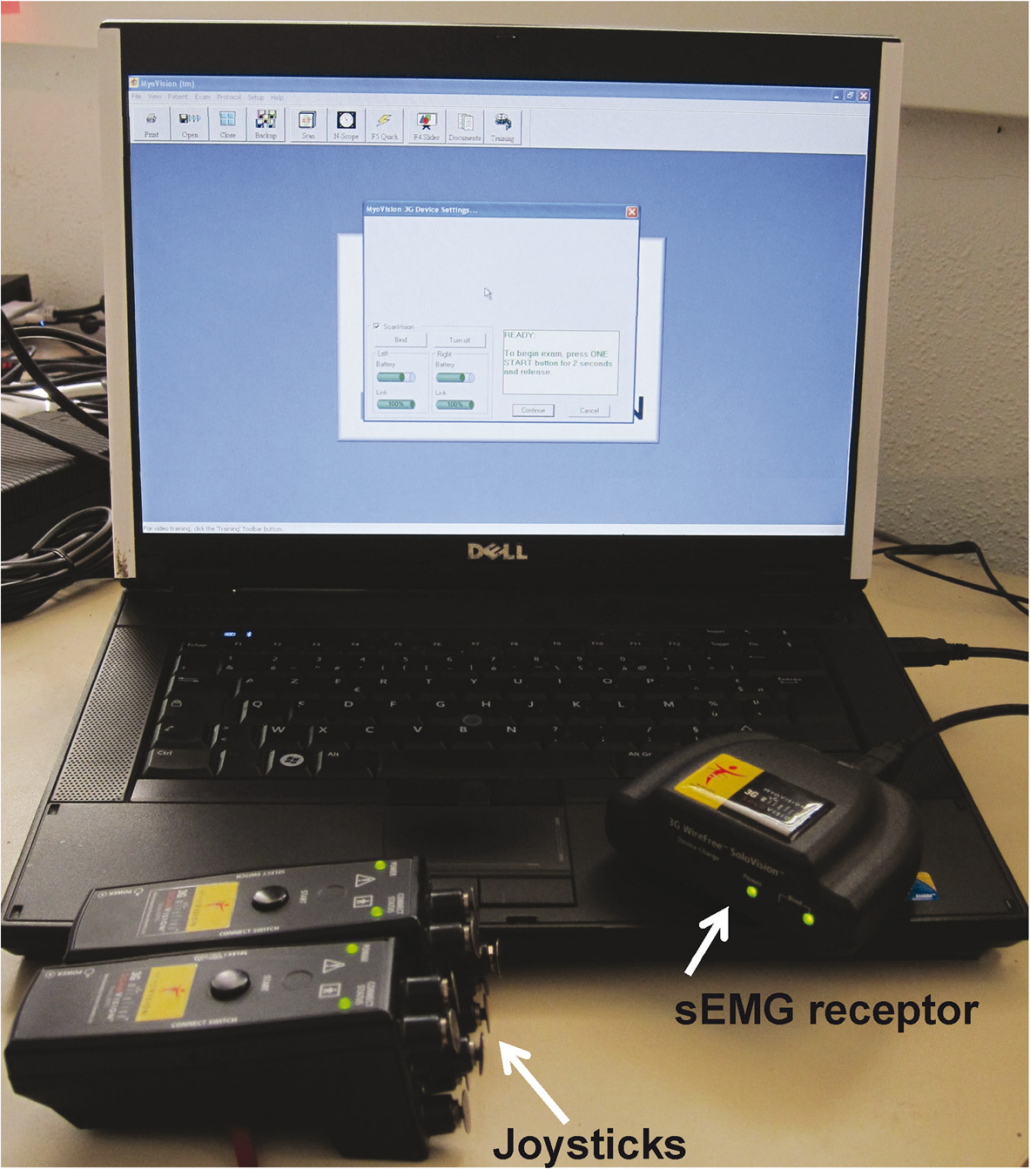
**Myovision® sEMG device.** The 2 joysticks are placed on both sides of the spine and data are recorded via the receptor linked to the computer.

**Figure 2:**
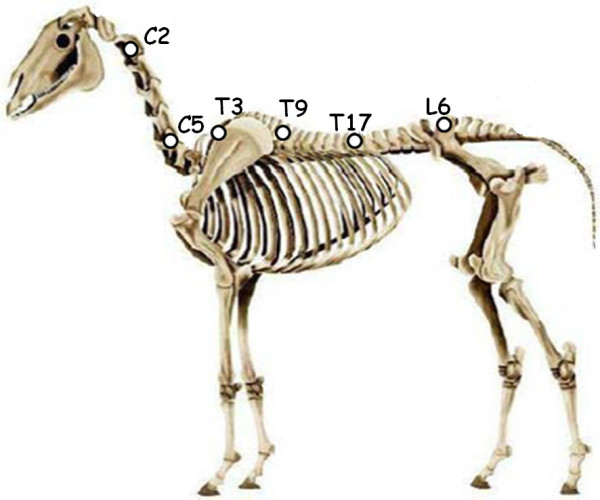
**Representation of a horse skeleton with the locations of electrodes for sEMG measurements.** The electrodes were placed at the level of the white spots of the figure (Adapted from Fureix et al [14]).

The sEMG examinations were conducted by the same experimenter (C.L), using a wire free device (Myovision®). The device was composed of 2 joysticks with 5 electrodes on each, designed to record muscle activities at the level of the vertebrae at the front and at the back of the joystick location. Muscular activities recorded were sent to a receptor connected to a computer (Figure 1) The joysticks were placed at the level of C2, C6, T3, T9, T17, and L6 (Figure 2) on both sides of the spine and the muscular activities at the level of C1, C3, C5, C7, T1, T3, T8, T10, T16, T18, L5 and S1 were recorded. Thus we obtained muscular activity all along the neck, at the level of the shoulder, at the base of the withers, at the level of the thoracolumbar joint and at the level of the lumbosacral joint, which are reported in the literature as very likely to be affected with spine lesions (*e.g.*[1,9]). The raw sEMG values were used (μV, see [28]). As it was previously shown to correlate with vertebral disorders [22], a vertebral site was considered as “affected” if the muscular activity at this level was over 10 μV in both sides of the spine. This 10 μV threshold was shown to relate to chronic vertebral disorders as evaluated by the practitioner in [22].

**Figure 3:**
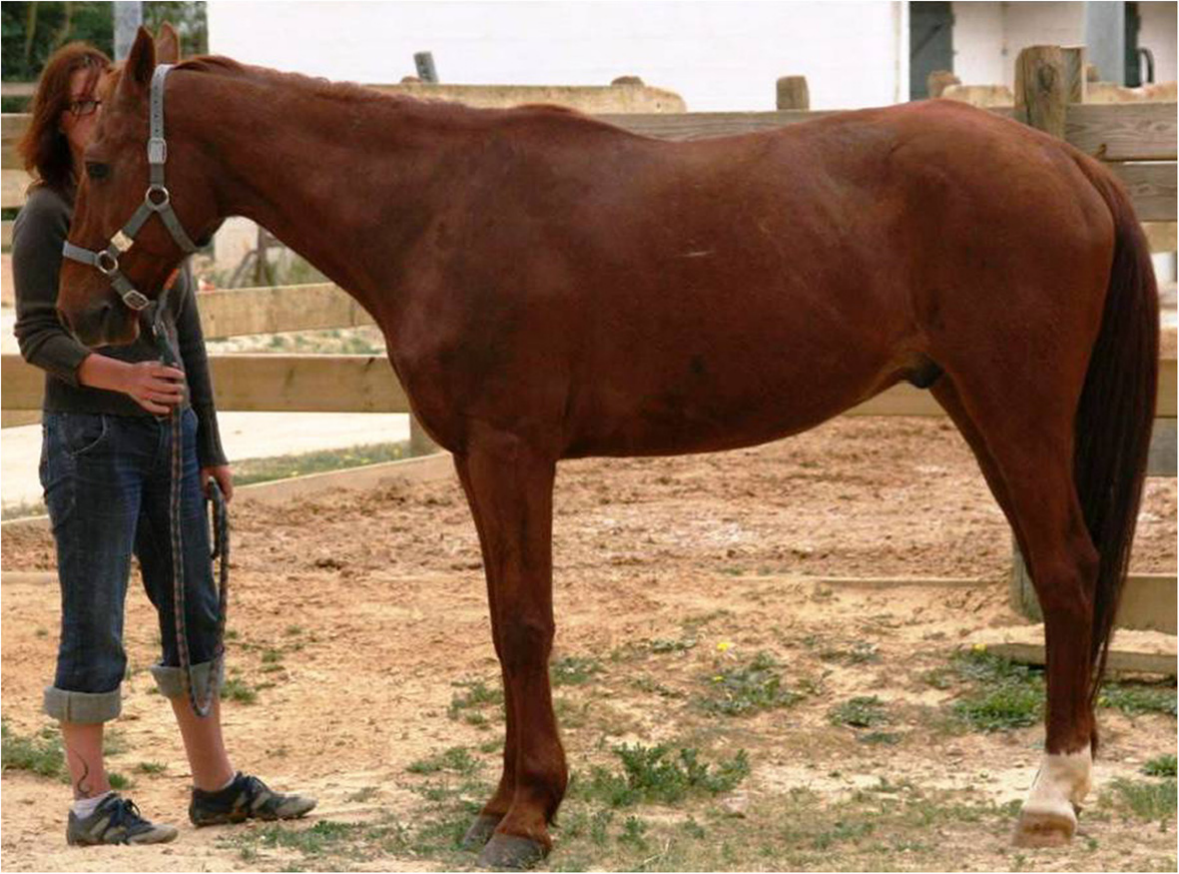
**Horses’ posture during sEMG examination.** The horse was slightly restrained with a halter and a rope on a flat ground, with anterior and posterior limbs placed in a line.

Examinations were performed on a flat ground, in the corridor of the stable in front of each horse’s box, without any noise or disturbance (working activity, people around…) to avoid any intrusive muscular mobilization. The experimenter paid attention to the horses’ feet position: anterior and posterior feet were aligned (Figure 3). Horses were kept motionless, slightly restrained.

### Questionnaire

The same questionnaire was given to caretakers for all horses (involved in Study 1 and in Study 2). In each riding school (N = 17), the person who was the most familiar to the horses (the caretaker involved in both daily and health care) was asked to answer a questionnaire about whether the horses in their care (N = 161) suffered chronic back pain, lameness, or any other chronic health problem during the past year (Table 1). Horses had been under the responsibility of this caretaker for at least 1 year. Questionnaires were given hand to hand, and were completed in every riding school for each horse. The caretakers were asked to tick boxes if their horses presented one of the listed chronic health problems. They were also asked to indicate any other chronic problem, especially those related to back pain or vertebral disorders they detected even if not listed in the questionnaire. The questionnaire was not specific to the evaluation of back pain problems, to evaluate whether, in a global context including all possible chronic disorders, back pain could be identified. Respondents were encouraged to report any supplementary comment they considered useful, concerning the possible causes of chronic disorders. However, if some caretakers actually wrote comments, the majority of them only ticked boxes. All respondents were able to see daily horses being ridden by riding schools’ pupils. They all graduated from French agricultural schools specialized in the horse industry, where they obtained a degree based on topics including basic notions on horse’s health, anatomy, physiology, care and management.

**Table 1:** Questionnaire given to the horses’ owners/caretakers

**Horse**	**Nothing to report**	**Lameness**	**Allergy**	**Cough**	**Ocular discharge**	**Sensitive to colic**	**Back pain**	**Stereotypy**		**Other chronic disorder**
								**Yes/No**	**Type of stereotypy**	
Horse 1										

The questionnaire included several possible chronic disorders and was not focused on back pain, in order to assess whether usual caretakers were aware to back pain amongst other chronic disorders. The questionnaire was completed in every school for every horse.

### Terminology

In order to simplify the understanding, we will define here the terminology used throughout the manuscript.

Chiropractic evaluation by manual palpation is efficient in the detection of muscular stiffness and vertebral mobility [15,16], and sEMG evaluation allows the detection of musculoskeletal dysfunctions [17,22]. All the disorders detected via manual palpation and sEMG evaluation have been grouped under “**back disorders**” throughout the manuscript (see also [1]).

### Statistical analyses

The number of subjects for each factor (site, sex, age) was unbalanced because of the availability of the different categories in the riding schools. Therefore, we used simple binomial GLM procedures without interactions which are known to be resistant, to assess the effects of site, sex and age in the 2 studies on the presence of back disorders. Our models were validated according to the usual validation procedure, using ANOVA and Chi square tests. Cohen’s kappa coefficient was used to test the agreement between the different evaluations and Chi Square tests were used to assess differences between clinical evaluations (manual palpation and sEMG measures) and questionnaires results. A significance threshold at p = 0.05 was used. All the statistics were completed using R software.

## Results

As a whole, this study was performed on 161 horses distributed across 17 riding schools all over France.

### Comparison between practitioner and questionnaire evaluations

#### Manual palpation

According to the manual palpation, 73% of the 59 horses were severely affected (at least 2 vertebral sites affected, N = 43), 12% were slightly affected (one vertebral site affected, N = 7) and only 15% were totally unaffected (N = 9). The proportion of horses displaying back disorders did not differ between schools (RidingSchool_1_ (RS1) = 100%, RidingSchool_2_ (RS2) = 93%, RidingSchool_3_ (RS3) = 66.7%; *χ*^2^ = 1.22, p = 0.54). However, neither age nor sex had any effect on the percentage of vertebral sites affected per horse detected with manual palpation (GLM, respectively F = 2.89 and F = 0.52, p = 0.09 and p = 0.47), but a strong site effect appeared (GLM, F = 4.45, p = 0.02) (*i.e.* from the raw data it was clear that there were as many horses with back disorders in RS3 as in the 2 others, but they had less vertebrae affected). Severely and slightly affected horses were pooled for further analyses (N = 50, 85% of the population).

#### Questionnaire evaluation

Out of the 59 horses, 22% (N = 13) were reported by owners/caretakers as having back pain. In this part of our study, none of the respondents reported anything, neither concerning possible causes, nor on the way back disorders were identified. Age had no effect on the prevalence of reported pain (GLM, p > 0.05). However, more mares were identified as being affected (47% mares affected N = 7/15, 14% geldings affected N = 6/44, GLM for binomial values, p = 0.005) and a strong riding school effect appeared, ranging from 0% to 58% of horses evaluated with back pain (RS_1_ = 50%, RS_2_ = 24%, RS_3_ = 0%; GLM, p = 0.001).

#### Comparison between manual palpation and questionnaire results

**Figure 4:**
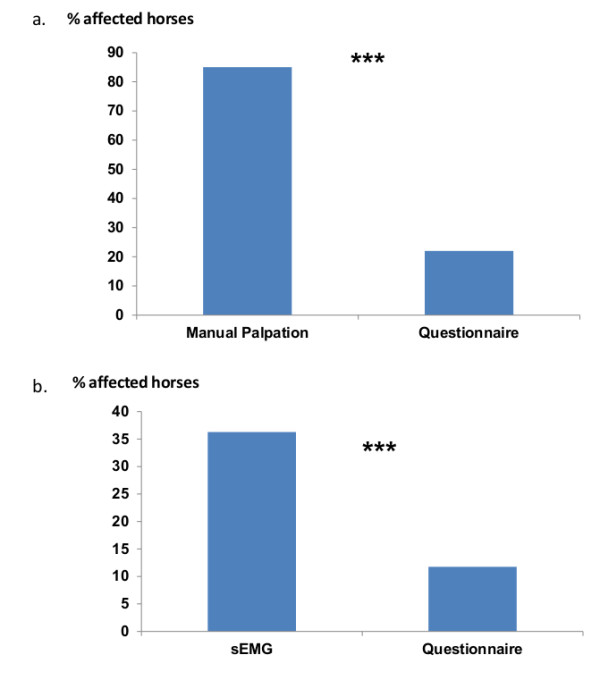
**Evaluation of back problems through clinical evaluations and questionnaires.** Note the similar discrepancies with questionnaires in both types of clinical evaluation. Chi square test, *** p < 0.001. **a)** Percentage of horses considered as affected via manual palpation on the left and questionnaire evaluation on the right. **b)** Percentage of horses considered as affected via sEMG on the left and questionnaire evaluations on the right.

The evaluations were in agreement for only 35.6% of the total population (number of horses with concordant evaluations: 13 affected and 8 healthy), leading to a poor Cohen’s kappa agreement coefficient evaluated at 0.09 (95% CI: 0-0.26). Evaluations were in agreement for only 50% of the RS1 horses (Kappa = 0, 95% CI: 0-0.57), 31% of the RS2 horses (Kappa = 0.04, 95% CI: 0-0.28) and 33% of the RS3 horses (Kappa = 0, 95% CI: 0-0.33). Moreover, the proportion of affected horses differed significantly between the 2 evaluation methods (Manual palpation = 46.8%, Questionnaire = 22%, *χ*^2^ = 49.3, p < 0.001) (Figure 4a). In all three sites, caretakers underestimated the prevalence of back disorders compared to the practitioner’s evaluation.

### Comparison between sEMG and questionnaire evaluations

#### sEMG evaluation

With the sEMG evaluation, 9 of the 14 riding schools had all their horses free from back disorders. At the population level, 36.3% (N = 37) of horses displayed high muscular activity at the level of at least 1 vertebral site tested. As in the previous study, neither sex nor age had any effect on the prevalence of back disorders in each horse (GLM for binomial values, respectively p = 0.28 and p = 24) or on sEMG measures (GLM, p = 0.43 and p = 0.65 respectively). Strong differences emerged between riding schools in the proportion of horses “affected” in at least one vertebral site (GLM for binomial values, p < 0.001), and in the proportion of vertebral sites affected in each horse (> 10 μV) ($\bar{X}$ per riding school: from 0% to 30.5%` vertebral sites affected per horse, GLM, F = 12.7, p < 0.001).

#### Questionnaire evaluation

Based on the questionnaire results, only 3.9% (N = 4/102) of horses were reported as having back pain. However, in this part of the study, three caretakers added comments on disorders associated with back pain, and 7.8% (N = 8/102) were reported as showing lameness with a possible back disorder cause. Horses considered as having back pain and lameness possibly associated with back disorders were pooled for subsequent analysis (N = 12, 11.8% in total). Neither sex nor age had any effect on the prevalence of back disorders reported by respondents (GLM, p = 0.30 and p = 0.19 respectively).

#### Comparison between sEMG and questionnaires results

In only 6 riding schools out of the 14 included in our study, respondents reported back pain (N = 3) or lameness associated with back pain (N = 3). As the number of back problems reported via questionnaire survey was too low, we could not compare the evaluation for each school. The Cohen’s kappa agreement coefficient between the two evaluations was very poor (Kappa = 0.08, 95% CI: 0-0.32). Moreover, the evaluation of back pain by caretakers was significantly lower than sEMG evaluation of back disorders (sEMG evaluation = 36.3%, Questionnaire = 11.8%, *χ*^2^ = 16.8, p < 0.001) (Figure 4b). Interestingly, out of the 12 horses reported by caretakers as suffering from back pain, only 6 presented high muscular activity, whereas 31 horses out of the 37 presenting high muscular activity at the level of at least 1 tested site were not reported as having back pain problems.

### Towards a riding school culture?

Strong differences appeared between the 17 schools in the prevalence of horses affected detected by clinical evaluations (*χ*^2^ = 47.8, p < 0.001) (Table 2), as well as in the detection of back disorders by caretakers (*χ*^2^ = 29.3, p = 0.02). However, “sound” versus “more affected” schools were highly different between back evaluations and questionnaires. In 4 of the 17 schools, caretakers over evaluated the prevalence of back problems compared to clinical evaluations. Interestingly, in these 4 riding schools, the proportion of affected horses was lower than in schools where caretakers did not report back pain (MW *U* test: U = 0, p = 0.002). On the contrary, in places where caretakers were confident that their horses did not have back pain, far more horses presented back disorders. These results highlight the high discrepancy between experimental evaluations and observations by daily caretakers.

**Table 2:** Proportion of “affected” horses per riding school as assessed by sEMG evaluation

**Riding school**	**RS4**	**RS5**	**RS6**	**RS7**	**RS8**	**RS9**	**RS10**	**RS11**	**RS12**	**RS13**	**RS14**	**RS15**	**RS16**	**RS17**
% of affected horses	0	100	71	100	30	0	0	0	14	0	0	0	0	0
Number of horses in the school	7	6	14	6	10	12	9	2	7	4	6	6	7	6

A horse was considered as “affected” as soon as the muscular activity at the level of one tested site was over 10 μV on both sides of the spine. The total number of tested horses in each school is also represented.

## Discussion

On the basis of both spine examination on the one hand and questionnaires to caretakers on the other, the estimated prevalence of back disorders in more than 150 riding school horses varied from 36.3% to 85% according to clinical examinations, but only from 3.9% to 22% according to questionnaire surveys. Thus, evaluations of back disorders by a practitioner (manual palpation) or with sEMG measures were in both cases higher than subjective evaluations by the familiar caretakers.

### Methodological considerations

Some differences were observed in the mean prevalence of back disorders between the first (chiropractor) and the second study (sEMG) which may be due to techniques (e.g. [15]) but also to the context: agricultural colleges with daily work in study 1 as opposed to traditional riding schools with 2 major working days in study 2.

#### Discrepancies between clinical and subjective evaluations

sEMG measures might be influenced by factors such as age, body fat, skin resistance or fear. In this study, horses all presented the same body condition (optimal), measures were conducted outside any disturbances and no fear reactions were observed. Furthermore, neither age nor breed had any effect on the muscular activity recorded, suggesting that if any of these parameters had an effect, it had to be minimal. We considered here only high muscular activity as reflecting back disorders, and further explorations are needed to investigate whether low or unbalanced muscular activity could also reflect a lack of musculature, and be a sign or a predictor of back disorders. However, high muscular activity has already been shown to be a good indicator of back disorders [22] and as they are easy to apply in field conditions, sEMG measures could be used more widely and on large samples of horses. In any case, whatever the type of evaluation, the prevalence obtained was much higher than that estimated by the caretakers’ responses to the questionnaires, and a former study showed that manual and sEMG evaluations were strongly consistent [22]. The low rate of back pain or disorders reported in the questionnaires confirms earlier results and highlights the difficulty of estimating/detecting/recognizing back pain problems in riding school horses [2,4]. Several reasons could explain the differences between the two clinical evaluations, as well as the discrepancies between clinical and questionnaire evaluations. 1) Horses show indirect or little expression of pain: increased aggression towards humans is often misinterpreted as “bad temper” [12], signs of escape attempts are sometimes misinterpreted as “bad willing” *e.g.*[5,29,30] and particular postures are often misread [14]. Behavioural problems (particularly aggressions) are a commonly reported source of accidents independently of the competency level of the person involved [31]. In addition, some postures reflecting back disorders have only recently been more thoroughly described (see [14,22,32]). Knowledge about the significance of escape behaviours during riding [13] or outside work [32] has not always reached professional caretakers. The present study underlines the urgent need for formal training to detect these signals. 2) Lack of attention may also be involved. Surveys have revealed that professionals’ risks of accidents with horses are more correlated with their exposure to horses (amount of time) than with a lack of experience, which can be explained by the reduced attention associated with increased routine [33,34]. Lowered attention may lead to neglect some signs of suffering. A questionnaire survey conducted on 3901 horses showed that the most commonly reported disorders were lameness (13% of the population) and skin disease (6.1% of the population), which are easily detectable because characterized by visible signs, whereas only 0.6% of the population was reported as suffering from back disorders [35]. A study conducted in United Kingdom also highlighted a very poor agreement between owners’ and veterinarians’ evaluation in geriatric horses’ health status [36]. Appropriate training, including learning to observe and being aware of postures and behaviours reflecting back disorders may help correct this failure in their detection. Thus, neck postures, both during [14] and outside [24] working time, increased aggressiveness [12] and overall postures [28,37] appear as potential indicators of the presence of back disorders. Moreover the awareness of more appropriate working [14] and living (Lesimple et al en prep) conditions may lead to a decrease of back disorders’ prevalence. 3) It is also possible that owners do not see or want to report these signs. Mills et al [38] suggested that owners could be unwilling to report “negative” elements about their animals. As one of the components of the welfare in animals is the absence of negative emotions [39], back pain can be considered as a serious welfare impairment (*e.g.*[8,12]) and it is possible that respondents balked at reporting such disorders. *In fine*, the low rate of back pain reports could be due to the very high prevalence of back disorders amongst riding school horses [12,14]: it is possible that animals with pain have become the norm, and that people do not discern external signs of pain anymore (see also Lesimple et al subm.). 4) Previous studies have shown that entire populations tend to show postural features that differ from other populations [28], which means that on a given site, all horses tend to show similar potential altered postures, that reflect an overall tendency for this site to have horses with back disorders (see also [14,24]). In such a case, “normality” for the local caretaker may be what the majority of horses on site express. Caretakers rarely have the opportunity to observe horses living for example in semi natural conditions.

### Towards better practices

As few people reported back pain in their horses, comparisons between each school could not be statistically tested. However, the differences emerging between the schools, both according to clinical and questionnaire evaluations highlight that people’s attention towards their horses is important. Indeed, caretakers who reported more horses suffering from back pain, sometimes even more than what was actually detected with clinical evaluations, were in riding schools in which horses were less affected. This might mean that in these schools people worried more about their horses’ general welfare (for example, horses spent more time in pasture, mostly in groups, Lesimple et al. in prep). It was previously shown that riding practices could greatly differ between schools and had an impact on horses’ back disorders [14]. Thus, one could think that in schools where the caretakers reported more back pain problems, people were more “sensitive” in a general way to their horses, thus promoting less constraining riding techniques as well as more positive environmental conditions. The absence of age effect on back disorders (sEMG and manual palpation evaluations) in our study, confirming earlier findings [1,3,9], might strengthen the hypothesis that environmental conditions, including working conditions, could be more important in the prevalence of back disorders than aging.

## Conclusions

This study is to our knowledge the very first to show that subjective evaluation, even by riding school owners or professional caretakers is not sufficient to evaluate back pain prevalence. As these problems are not detected, horses suffering from back pain or disorders may keep on working, leading to a possible worsening of the situation. Moreover, even if further investigations are needed to assess whether low or unbalanced muscular activity could also be a sign of vertebral or back pain problems, sEMG measures are efficient in the detection of the presence of back disorders [24], and can be used easily and efficiently in field conditions on large samples of horses. Training professionals to pay more attention to horses’ postures and behaviours reflecting back disorders and increasing their awareness of the problem could lead to a questioning around horses’ welfare in general, and as a consequence, to improved environmental conditions of horses.

## Consent

The person present on the Figure 3 is the first author of this paper and gave her consent for the publication of the picture.

## Competing interests

The authors declare no competing interests. All the manual palpations were performed for free by H. Menguy himself, manager and only employee of the chiropractic practice. Moreover the manual palpations were carried on Sunday, outside working time of the practice.

## Authors’ contributions

MH, CL and CF conceived and designed the experiments, CL CF and HM performed the experiments, CL, VB and MH analyzed the data. CL and MH wrote the paper. All authors read and approved the final manuscript.
